# Evaluation of Natural Polyphenols Encapsulated in Multiparticulate Drug Delivery Systems as Potential Therapeutics for Cutaneous Melanoma

**DOI:** 10.3390/jfb17070317

**Published:** 2026-07-01

**Authors:** Andreea D. Lazar (Popa), Mădălina G. Albu-Kaya, Sorina Dinescu

**Affiliations:** 1 Department of Biochemistry and Molecular Biology, University of Bucharest, 050095 Bucharest, Romania; andreea.lazar@bio.unibuc.ro; 2 Division of Leather and Footwear Research Institute, National Research and Development Institute for Textiles and Leather, 93 Ion Minulescu Str., 031215 Bucharest, Romania; albu_mada@yahoo.com; 3 Research Institute of the University of Bucharest (ICUB), 050663 Bucharest, Romania

**Keywords:** cutaneous melanoma, curcumin, quercetin, multiparticulate drug delivery systems, chemoprevention

## Abstract

Nutraceuticals are bioactive compounds that can contribute to maintaining general health and supporting the treatment of various conditions. Among them, flavonoids such as curcumin (C) and quercetin (Q) are of particular interest in oncology research, including melanoma, due to their anti-tumor effects. However, due to poor bioavailability, the development of efficient delivery vehicles is of utmost importance. In this context, we aimed to assess the effectiveness of a potential treatment on the progression of cutaneous melanoma by evaluating cell viability and proliferation in the presence of encapsulated C/Q in innovative microcapsules (MDDS) embedded in a collagen matrix, as well as the anti-inflammatory and antioxidant potential. We found that tumor cell viability and proliferation were significantly reduced in the presence of C/Q, with the best results being obtained for the composite with both bioactive compounds, these results were also supported by a significant increase in pro-apoptotic markers. A decrease in pro-inflammatory markers was observed, as well as a decrease in ROS secretion over time, indicating the anti-inflammatory and antioxidant potential of the materials supplemented with the tested flavonoids, these properties being important for TME modulation. Therefore, the MDDS-CQ embedded sponge could represent a potential adjuvant therapy for cutaneous melanoma in the future.

## 1. Introduction

Malignant melanoma is an aggressive neoplasm responsible for over 75% of skin cancer-related deaths. Its incidence has risen worldwide faster than that of any other cancer, compounded by its high metastatic potential and limited response to current systemic therapies. Once metastatic disease develops, the prognosis remains poor, with a 5-year survival rate of only 15–20% and a median survival of 8–12 months. These sobering outcomes underscore the urgent need for effective preventive and early-intervention strategies, as well as novel strategies that improve therapeutic delivery, enhance efficacy, and reduce systemic toxicity [1,2,3,4].

Chemoprevention, defined as the use of pharmacological or natural agents to inhibit cancer initiation, promotion, and progression, has been an active field of research in recent decades. Among the most extensively investigated chemopreventive agents are phytocompounds—biologically active molecules abundant in fruits, vegetables, roots, and herbs, such as curcumin and quercetin—which exhibit antioxidant, anti-inflammatory, and anti-tumor properties by modulating signaling pathways involved in cell proliferation, apoptosis, angiogenesis, and metastasis [5,6,7]. Curcumin, the principal bioactive compound of *Curcuma longa*, exerts anti-proliferative and anti-inflammatory effects through multiple mechanisms, including inhibition of NF-κB signaling, induction of apoptosis via caspases, downregulation of cyclin D1, and suppression of epithelial–mesenchymal transition. These multifaceted effects have supported its evaluation in early-phase clinical trials for chemoprevention [8,9,10,11]. Quercetin, a widely distributed dietary flavonoid, regulates oxidative stress, apoptosis, and metastasis, acting as an antioxidant at low concentrations and selectively inducing ROS-mediated cytotoxicity in tumor cells at higher doses [12,13,14,15,16,17,18]. In combination, curcumin and quercetin demonstrate synergistic anti-tumor effects, enhancing proliferation inhibition and modulating apoptotic and Wnt/β-catenin signaling pathways. Collectively, the literature supports the potential of these phytocompounds—both individually and in combination—as promising agents for melanoma chemoprevention, owing to their complementary antioxidant, immunomodulatory, and cytotoxic properties [19].

Despite promising preclinical evidence, the therapeutic potential of curcumin and quercetin is constrained by their physicochemical limitations, most notably their poor aqueous solubility. This low solubility translates into limited dissolution in physiological fluids, resulting in poor bioavailability, rapid metabolism, and inconsistent systemic exposure. Additionally, both compounds exhibit chemical instability under physiological conditions, further diminishing their effective concentration at target sites. These constraints necessitate relatively high doses to achieve measurable effects, which can increase variability and reduce translational reliability. Nonetheless, these same limitations highlight substantial room for optimization. Advances in formulation strategies, such as encapsulation (micro/nanoparticles), solid dispersions, phospholipid complexes, and co-crystallization, have demonstrated the potential to markedly enhance solubility, chemical stability, sustained release, and absorption, improving local bioavailability while minimizing systemic exposure. Scaffold-based delivery, in particular, provides a platform for maintaining effective concentrations of phytocompounds at the tumor site or in tissue models, representing a promising approach for adjuvant or chemopreventive therapy [20,21,22,23].

Therefore, to achieve the maximum therapeutic potential of curcumin and quercetin, it is necessary to develop new delivery systems for the controlled and localized release of these two flavonoids, one example being polymeric microcapsules. Biodegradable natural polymers, such as collagen, gelatin, alginate, and cellulose, are considered ideal vehicles for drug delivery. Although their mechanical properties and resistance to enzymatic degradation in vivo are not ideal, these characteristics can be improved by cross-linking, using various cross-linking agents [24]. Finally, the selection of biocompatible materials to be used as carriers for curcumin and quercetin will be key to the success of these new therapeutic approaches for chemoprevention.

In this study, we investigated the effect of curcumin and quercetin, encapsulated in innovative Multiparticulate Drug Delivery Systems (MDDS), on primary melanoma cells with invasive tendencies, in order to highlight their efficiency as potential inhibitors of tumor progression.

## 2. Materials and Methods

### 2.1. Experimental Models

Tumor cells from the primary melanoma cell line MelJuSo (ACC-74, DSMZ) were cultured in RPMI-1640 medium (Sigma Aldrich Co., Steinheim, Germany) supplemented with 10% fetal bovine serum (FBS) and 1% antibiotic–antimycotic solution. Cells were maintained under standard culture conditions at 37 °C in a humidified atmosphere containing 5% CO_2_, with the culture medium being refreshed every 2–3 days.

Materials were synthesized and characterized in our previous study [25]. Briefly, the microcapsule–collagen gel system consisted of 30% (*w*/*v*) microcapsules loaded with curcumin (0.08%, *w*/*w*) and/or quercetin (0.04%, *w*/*w*) incorporated into a collagen matrix.

Details regarding the composition of the MDDS and collagen-based materials are presented below (Table 1 and Table 2).

Gel pieces (~1 cm², ~1 mm thickness) were applied to each well, corresponding to nominal amounts of 24 µg curcumin and 12 µg quercetin per piece. The system provides sustained release of the compounds over the experimental period (based on previously reported characterization of microcapsule composition and release kinetics; Reference: [26]). Based on the release profile (~80% compound liberation after 48 h), the effective concentrations available in vitro were ~50 µM curcumin and ~30 µM quercetin. Preliminary IC50 tests of free curcumin and quercetin were performed to guide the concentration range used in the current study. For long-term treatments (≥72 h), culture medium was not replaced or supplemented, allowing continuous exposure of cells to the compounds released from the microcapsules.

### 2.2. Physicochemical Characterization and Release Performance of Collagen-Based Scaffolds Incorporating Microcapsules

Regarding the physicochemical characterization and release performance of the collagen-based scaffolds incorporating polymeric microcapsules (alginate/carboxymethyl cellulose/gelatin), our previous studies investigated them as localized delivery systems for wound healing applications [25,26]. The systems employed microcapsules embedded within a collagen-rich matrix and produced by freeze-drying. Investigations demonstrated that incorporation of microcapsules substantially altered the physicochemical properties of the collagen scaffolds. The presence of microcapsules consistently reduced scaffold swelling capacity relative to capsule-free controls, after 24 h, water uptake decreased from approximately 40 g/g for the control material to 22.7–33.2 g/g in microcapsule-containing formulations, depending on flavonoid composition, indicating that the swollen capsules generated a denser and less porous architecture. Microcapsule incorporation also enhanced scaffold resistance to enzymatic degradation. In the collagen-based composites, degradation of the control scaffold reached approximately 96% after six days, while flavonoid-loaded microcapsule formulations showed substantially improved stability, with degradation values of 37.2%, 57.4%, and 15.8% for Coll-C, Coll-Q, and Coll-CQ, respectively. Morphological analysis further confirmed the influence of microcapsules on scaffold architecture. The collagen-based composites displayed highly porous and interconnected structures with embedded microcapsules distributed throughout the matrix. Pore sizes ranged from 50 to 100 μm, a range considered favorable for cellular infiltration and skin tissue regeneration. FTIR analysis additionally demonstrated preservation of the collagen structural features and suggested that the microcapsules remained intact throughout the freeze-drying process. Considering the release kinetics, microcapsule-loaded scaffolds exhibited a characteristic biphasic release profile consisting of an initial burst release followed by sustained drug delivery. Encapsulation significantly reduced the burst effect, decreasing first-hour release from 39.5% in the non-encapsulated scaffold to 12.3–19.7% in microcapsule-containing formulations. Sustained release continued for up to 48 h, achieving cumulative release values between 76.2% and 95.0%, depending on scaffold composition. Release data were best described by the Power Law (Korsmeyer–Peppas) model, indicating a non-Fickian transport mechanism governed by a combination of diffusion, polymer relaxation, swelling, and matrix degradation processes. Overall, our studies demonstrated that incorporation of polymeric microcapsules into collagen-based scaffolds improves structural stability and modulates scaffold hydration behavior while maintaining a highly porous architecture. Furthermore, microencapsulation enables controlled delivery of bioactive agents and significantly reduces burst release, highlighting the potential of these collagen-based systems not only for wound healing but also for other applications where the delivery of flavonoids is desired [25,26].

### 2.3. Quantitative and Qualitative Assessment of Cell Viability, Proliferation, and Cytotoxicity

Cytocompatibility tests were performed in order to highlight the effect of the flavonoids on cell viability and proliferation. In this regard, cutaneous tumor cells from the MelJuSo cell line were thawed and maintained in culture. The 3D materials were sterilized by UV exposure for 6 h, on both sides, and washed with SFB-free medium and 5% antibiotic to remove any toxic products remaining from the synthesis of the materials. Subsequently, they were swollen in culture medium and cut to a size of 1 cm^2^. The prepared materials were placed in 48-well plates and seeded with 2 × 10^5^ cells/cm^2^. After seeding, the plates were placed in an incubator and kept under standard culture conditions (37 °C, 5% CO_2_, humidity), for three and seven days, respectively. Quantitative spectrophotometric (MTT, LDH) and qualitative (Live/Dead fluorescence microscopy) cytocompatibility tests were performed on the in vivo-like experimental models.

The viability and proliferation of melanoma cells cultured in contact with the tested materials were evaluated using the MTT assay (methylthiazolyldiphenyl tetrazolium bromide; Sigma-Aldrich, Steinheim, Germany). The MTT reagent was prepared in phosphate-buffered saline (PBS) at a concentration of 1 mg/mL, following the manufacturer’s protocol. Samples were incubated with the MTT solution for 4 h under standard culture conditions (37 °C, 5% CO_2_, humidified atmosphere) to allow the formation of formazan crystals by metabolically active cells. Following incubation, the crystals were dissolved in isopropanol, and the absorbance of the resulting solution was measured at 550 nm using a FlexStation 3 spectrophotometer (Molecular Devices, San Jose, CA, USA). The absorbance values were used as an indicator of the number of viable, metabolically active cells.

The cytotoxicity of the tested materials was assessed using a lactate dehydrogenase (LDH) assay (TOX7-1KT, In Vitro Toxicology Assay Kit, LDH-Based; Sigma/Merck, Steinheim, Germany). The LDH reaction mixture was prepared according to the manufacturer’s instructions and added to the collected culture supernatants in a 96-well plate. Samples were incubated for 15 min at room temperature in the dark to allow color development. LDH released into the culture medium by cells with compromised membrane integrity was quantified by measuring the absorbance of the reaction product at 490 nm using a FlexStation 3 spectrophotometer (Molecular Devices, San Jose, CA, USA). The absorbance values were considered proportional to the extent of cellular membrane damage and cytotoxicity.

Cell viability following contact with the tested materials was assessed using a Live/Dead assay (Invitrogen, Life Technologies, Foster City, CA, USA). The test was based on the simultaneous staining of viable cells with calcein acetoxymethyl ester (calcein AM, green fluorescence) and non-viable cells with ethidium homodimer-1 (EthD-1, red fluorescence). The staining solution was prepared according to the manufacturer’s instructions, added to the samples, and incubated for 2 h at room temperature in the dark. Subsequently, the cells were examined using a Nikon A1/A1R laser-scanning confocal microscope (Nikon Instruments Inc., Melville, NY, USA). Acquired images were analyzed using NIS-Elements software (version 5.11, 64-bit; Nikon Instruments Inc., Melville, NY, USA) to evaluate cell viability and distribution.

### 2.4. Gene Expression Evaluation of Apoptotic Markers by qRT-PCR and Statistics

To investigate the effect of the flavonoids C and/or Q, encapsulated and embedded in collagen-based materials (in vivo-like model), on cell survival, the gene expression of markers involved in apoptosis (Bax, Bcl-2, Caspase-3) was evaluated by qPCR. Comparisons were made between melanoma cells subjected to the treatment for 24 h (conditioned culture medium enriched with C/Q extract released from the materials over 72 h), and the untreated condition. For gene expression analyses, conditioned medium obtained from the microcapsule–collagen constructs was used instead of direct scaffold-contact cultures in order to evaluate the effects of the released compounds independently of scaffold–cell interactions and to facilitate standardized cell recovery and high-quality RNA isolation for downstream molecular analyses. Following treatment with conditioned medium released from the microcapsule–collagen gels, cells were harvested by trypsinization, washed twice with PBS, and counted to ensure standardized RNA input. Total RNA was extracted using TRIzol reagent (Thermo Fisher Scientific Waltham, MA, USA) according to the manufacturer’s instructions. RNA concentration and purity were determined spectrophotometrically using a NanoDrop 8000 spectrophotometer (Thermo Scientific, Waltham, MA, USA). Complementary DNA (cDNA) was synthesized from the isolated RNA using the iScript cDNA Synthesis Kit (Bio-Rad, Hercules, CA, USA) and a Veriti 96-Well Thermal Cycler (Applied Biosystems, Waltham, MA, USA). Quantitative real-time PCR (qPCR) was subsequently performed on a ViiA™ 7 Real-Time PCR System (Applied Biosystems) using Forget-Me-Not™ EvaGreen^®^ qPCR Master Mix (Biotium, Fremont, CA, USA) and gene-specific primers. Relative gene expression levels were normalized to the housekeeping gene GAPDH. Statistical analyses were conducted using GraphPad Prism software (version 9.0). Data are presented as mean ± standard deviation (SD). Differences among groups were evaluated by one-way analysis of variance (ANOVA) followed by Bonferroni’s multiple-comparison test, with *p* < 0.05 considered statistically significant.

### 2.5. Protein Expression Evaluation of Apoptotic Markers by Immunolabeling and Confocal Microscopy

Tumor cells were seeded on Coll materials, supplemented or not with flavonoids, at a density of 2 × 10^5^ cells/cm^2^, and maintained for 24 h under standard culture conditions. The expression of the pro-apoptotic proteins Bax and cleaved Caspase-3 was evaluated by immunofluorescence staining followed by confocal microscopy analysis. Cells were fixed with 4% paraformaldehyde (Sigma-Aldrich) for 2 h and subsequently permeabilized and blocked using a solution containing 0.1% Triton X-100 and 2% bovine serum albumin (BSA; Sigma-Aldrich) for 1 h at room temperature. Samples were then incubated overnight at 4 °C with primary antibodies against Bax (mouse monoclonal antibody, Santa Cruz Biotechnology, Dallas, TX, USA; 1:100 dilution) and cleaved Caspase-3 (Asp175) (rabbit monoclonal antibody, Cell Signaling Technology, Danvers, MA, USA; 1:1000 dilution). Following primary antibody incubation, cells were washed and incubated for 2 h at room temperature in the dark with Alexa Fluor 488-conjugated goat anti-mouse IgG and Alexa Fluor 546-conjugated goat anti-rabbit IgG secondary antibodies (Invitrogen, Waltham, MA, USA), both diluted 1:500. Cell nuclei were counterstained with Hoechst 33342 solution (Thermo Fisher Scientific) for 15 min at room temperature in the dark. Fluorescence imaging was performed using a Nikon AX R Eclipse Ti2-E confocal microscope (Nikon Instruments Inc., Melville, NY, USA). Image acquisition and analysis were carried out using NIS-Elements software (version 5.21, 64-bit; Nikon Instruments Inc., Melville, NY, USA).

### 2.6. Evaluation of the Anti-Inflammatory Effect of Coll-MDDS Complexes Embedded in Materials

Melanoma cells from the MelJuSo cell line were cultured under standard conditions to investigate the anti-inflammatory potential of the developed biomaterials. The expression of the pro-inflammatory cytokines interleukin-6 (IL-6) and tumor necrosis factor-α (TNF-α) was evaluated at both the gene and protein levels 48 h after cell seeding. Scaffolds composed of collagen without flavonoid incorporation (Coll) served as the negative control, while cells cultured directly on tissue culture plastic served as the positive control. Gene expression analysis was performed by quantitative real-time PCR (qPCR) using 2× Forget-Me-Not™ EvaGreen^®^ qPCR Master Mix and a ViiA™ 7 Real-Time PCR System (Applied Biosystems). Protein expression was assessed by immunofluorescence staining and confocal microscopy following the protocol described in Section 2.5, with the addition of an F-actin staining step using phalloidin-FITC solution (Sigma-Aldrich, Steinheim, Germany) for 1 h at room temperature in the dark. Primary antibodies against IL-6 and TNF-α (Proteintech, Planegg-Martinsried, Germany) were used at a dilution of 1:300. The secretion of IL-6 and TNF-α into the culture medium was further quantified 48 h after melanoma cells were cultured in contact with the collagen-based scaffolds using an enzyme-linked immunosorbent assay (ELISA; Qiagen, Hilden, Germany), according to the manufacturer’s instructions. Absorbance was measured at 450 nm using a FlexStation 3 microplate reader (Molecular Devices, San Jose, CA, USA). Data visualization and statistical analyses were performed using GraphPad Prism software.

### 2.7. Evaluation of the Antioxidant Effect of Flavonoids Encapsulated in the Materials

To evaluate the antioxidant effect of curcumin (C)- and/or quercetin (Q)-enriched composites, MelJuSo melanoma cells were cultured under standard conditions and placed in contact with the microcapsule–collagen gel materials. Intracellular reactive oxygen species (ROS) production, specifically hydrogen peroxide (H_2_O_2_), was quantified fluorometrically at 24, 48, and 72 h after cell seeding using the Amplex™ Red Hydrogen Peroxide/Peroxidase Assay Kit (10-acetyl-3,7-dihydroxyphenoxazine; Cat. No. A22188, Thermo Fisher Scientific, Waltham, MA, USA). The assay was performed according to the manufacturer’s instructions, and fluorescence intensity was measured as an indicator of H_2_O_2_ generation. For sample preparation, at each time point, culture medium in contact with the materials was collected and directly analyzed for H_2_O_2_. No cell lysis was performed, as the assay measures extracellular H_2_O_2_ released by the cells. A control sample consisting of melanoma cells grown on plastic and treated with H_2_O_2_ was considered a positive reference to confirm assay performance, whereas the RPMI-1640 culture medium without cells or treatment was considered a negative control. Fluorescence detection was performed at the excitation/emission wavelengths specified by the manufacturer. Data were expressed as mean ± SD of three independent experiments.

### 2.8. Fluorescence Quantification of Cell Imaging

Fluorescence intensity associated with the expression of apoptotic and anti-inflammatory markers was quantified using ImageJ software (version 1.x bundled with 64-bit Java 8). Data processing, graphical representation, and statistical analyses were performed using GraphPad Prism software (version 9.0; GraphPad Software Inc., San Diego, CA, USA). Statistical significance was evaluated by one-way analysis of variance (ANOVA) followed by Bonferroni’s post hoc multiple-comparison test. Results are presented as mean ± standard deviation (SD) calculated from 10 fields of view per sample (*n* = 10). Differences were considered statistically significant at *p* < 0.05.

### 2.9. Statistical Analysis

All experiments were performed using three independent biological replicates (*n* = 3). For quantitative assays (MTT, LDH, qPCR, ELISA, and ROS measurements), data from each biological replicate were included in the statistical analysis and are presented as mean ± SD. For immunofluorescence and fluorescence quantification analyses, 10 randomly selected microscopic fields of view were acquired from each sample. Fluorescence measurements from these fields were averaged to obtain a single value for each biological replicate prior to statistical analysis. Data were analyzed and graphically represented using GraphPad Prism software (version 9.0; GraphPad Software Inc., San Diego, CA, USA). Statistical significance was determined by one-way analysis of variance (ANOVA) followed by Bonferroni’s multiple-comparison correction post-test. Differences were considered statistically significant when *p* < 0.05.

## 3. Results

Three-dimensional collagen-based materials (Coll), supplemented with flavonoids encapsulated in innovative microcapsules: curcumin (Coll-C), quercetin (Coll-Q) or both (Coll-CQ), were evaluated in terms of their effects on tumor cell viability and proliferation, anti-inflammatory and antioxidant potential.

### 3.1. Quantitative and Qualitative Assessment of Cell Viability, Proliferation, and Cytotoxicity

Cell viability and proliferation were evaluated by quantitative cytocompatibility tests (MTT, LDH) and qualitative assay (LiveDead) on in vivo-like experimental models (resulted from the seeding of tumor cells from the primary melanoma cell line MelJuSo on the three-dimensional sponges Coll, Coll-C, Coll-Q and Coll-CQ), at two time points, specifically 3 and 7 days post-seeding.

**Figure 1:**
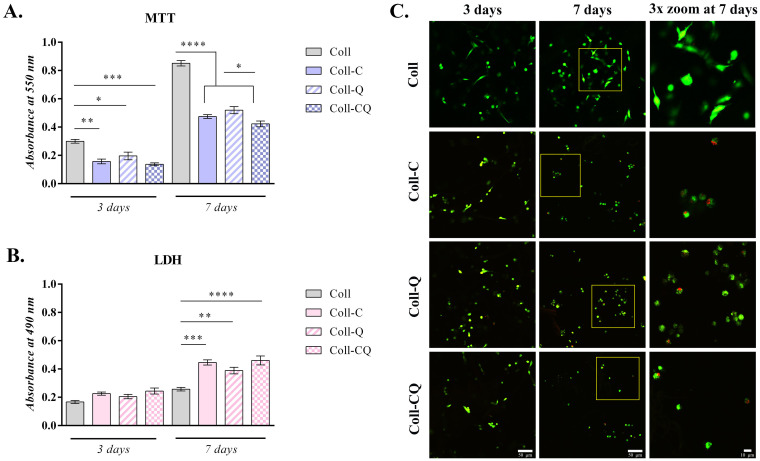
Quantitative and qualitative assessment of cell viability, proliferation, and cytotoxicity after 3 and 7 days of melanoma cell culture in contact with the delivery systems: (**A**) Evaluation of melanoma cell viability and proliferation in the presence of encapsulated curcumin and/or quercetin using the MTT assay; (**B**) Cytotoxicity of MDDS-C/Q complexes evaluated by the quantitative LDH assay; (**C**) Cell viability assessment by LiveDead assay indicating live cells (labeled with calcein–green) and dead cell nuclei (ethidium homodimer–red) for melanoma cells exposed to MDDS-C/Q. Scale bars 50 μm and 10 μm (3× magnification). The yellow square indicates the region selected for magnification. Statistical significance: * *p* < 0.05, ** *p* < 0.01, *** *p* < 0.001, **** *p* < 0.0001 versus the Coll control group. Additional comparisons between flavonoid-loaded groups are indicated by connecting lines and corresponding *p*-values. Specific dosages of natural agents released from MDDS: 50 µM curcumin and 30 µM quercetin.

Results obtained from the quantitative assessment revealed that the tested materials enriched with C and/or Q decreased melanoma cell viability and proliferation. As shown in Figure 1A, the viability level was significantly reduced in the case of melanoma cells exposed to the flavonoid-enriched composites compared to the Coll control, at both time points after treatment initiation. According to the MTT test, after 3 days, cell viability decreased significantly in the three materials supplemented with flavonoids (*p* = 0.0062 for Coll-C, *p* = 0.0363 for Coll-Q, *p* = 0.004 for Coll-CQ), the best result being obtained for the composite enriched with both bioactive compounds. At 7 days, all C/Q materials showed a statistically significant decrease in cell viability compared to the Coll control (*p* < 0.0001), with a difference even between the Coll-Q and Coll-CQ materials (*p* = 0.0284), suggesting an enhanced anti-tumor effect of the combined flavonoid treatment compared with either compound alone.

The LDH assay indicated the levels of cytotoxicity induced by the exposure of melanoma cells to the C/Q complexes embedded in the materials. The results were consistent with those obtained by the MTT assay (Figure 1B). Thus, a higher level of cytotoxicity was observed in the case of materials enriched with flavonoids, compared to the Coll control. Although at 3 days, the differences recorded are not statistically significant, after 7 days of treatment, a significant increase in cytotoxicity was observed for Coll-C (*p* = 0.0003), Coll-Q (*p* = 0.0072) and Coll-CQ (*p* < 0.0001), the largest difference being found for the material supplemented with both nutraceutical compounds, which suggests their cumulative anti-tumor effect.

The LiveDead assay allowed simultaneous fluorescent labeling of live cells (green) and dead cell nuclei (red) in the case of the primary melanoma cell line MelJuSo exposed to collagen-based composites. As shown in Figure 1C, a lower viability can be observed in the presence of MDDS-C/Q, compared to the control material, both at 3 days and 7 days after the initiation of treatment. If at 3 days, the anti-tumor effect is not as pronounced, at 7 days significant cytotoxic effects are evident for all C/Q-enriched composites, especially Coll-CQ, most cells had red nuclei, suggesting their lack of viability. At the same time, while at the level of Coll, a significant increase in cell proliferation was recorded from 3 to 7 days, in the case of MDDS-C/Q-sponges this increase is not visible.

### 3.2. Evaluation of Anti-Tumor Effect of MDDS-C/Q on Specific Markers Involved in Cell Death

The anti-tumor effect of flavonoids C and/or Q encapsulated and embedded in collagen-based materials was determined by analyzing the gene and protein expression of specific markers involved in cell death, in melanoma cells subjected to the treatment, compared to the untreated condition.

**Figure 2:**
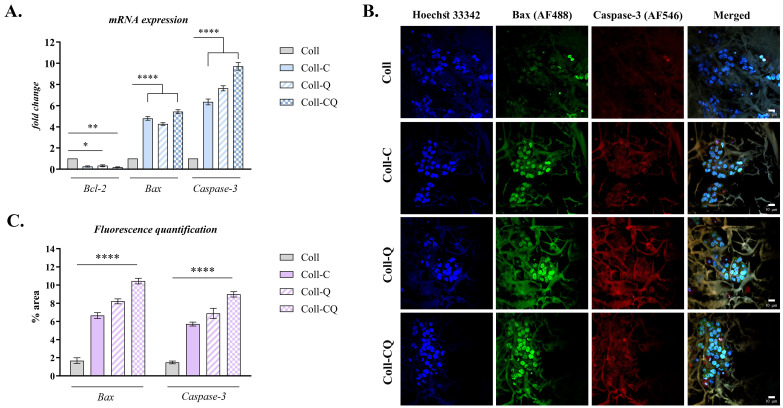
Evaluation of the anti-tumor effect of the delivery systems on specific markers involved in cell death: (**A**) Gene expression profiles of pro-apoptotic markers Bax and Caspase-3 and the anti-apoptotic marker Bcl-2, compared between melanoma cells treated for 24 h with conditioned culture medium obtained after 72 h from the materials enriched with C/Q (Coll-C, Coll-Q, Coll-CQ) and the simple collagen-based control (Coll). Data are normalized to GAPDH expression level. Statistical significance: * *p* < 0.05, ** *p* < 0.01, **** *p* < 0.0001 versus the Coll control group; (**B**) Fluorescence microscopy images showing the expression of the pro-apoptotic protein BAX labeled with Alexa Fluor 488 antibody (green), the pro-apoptotic protein Caspase-3 labeled with Alexa Fluor 546 antibody (red), and nuclei with Hoechst (blue) in tumor cells seeded on the tested materials, obtained with the 60× objective. Scale bar 10 μm. (**C**) Quantification of green fluorescence (protein expression of Bax, labeled AF488) levels and red fluorescence (protein expression of Caspase-3, labeled AF546) levels in all composites. Statistical significance: *p* < 0.0001 (****). Specific dosages of natural agents released from MDDS: 50 µM curcumin and 30 µM quercetin.

Gene expression analysis of Bax, Caspase-3 and Bcl-2 (Figure 2A), markers involved in apoptosis, revealed that the exposure of melanoma cells to encapsulated flavonoids significantly affected their expression, with a statistically significant increase in the expression of pro-apoptotic markers Bax and Caspase-3 (*p* < 0.0001 for all composites with C and/or Q), as well as a statistically significant reduction in the expression of the anti-apoptotic marker Bcl-2 (*p* < 0.032 for C/Q and *p* < 0.0028 for Coll-CQ) in cells treated with MDDS-C/Q compared to the control (Coll), which suggests that the materials with C/Q induce tumor cell death through the phenomenon of apoptosis, and the presence of both nutraceutical compounds potentiates the anti-tumor effect.

The protein expression of pro-apoptotic markers Bax and Caspase-3, was evaluated by immunolabeling coupled with fluorescence microscopy, in the case of melanoma cells exposed to the MDDS-C/Q complexes, embedded in the collagen matrix, with similar results. Thus, as can be seen in Figure 2B,C, the expression of the pro-apoptotic proteins Bax and Caspase-3 significantly increased in tumor cells found in contact with the C and/or Q-enriched sponges (approximately 6× higher for both markers in the case of Coll-C, 7–8× higher for Bax and Caspase-3 in melanoma cells subjected to Coll-Q), the best result being obtained for the composite with both bioactive compounds (Coll-CQ, almost 10× higher), suggesting that the findings are consistent with apoptosis-mediated cell death.

### 3.3. Evaluation of Anti-Inflammatory Potential of MDDS-C/Q

The anti-inflammatory potential of C/Q flavonoids encapsulated in MDDS and embedded in collagen matrices (Coll) was evaluated comparatively by analyzing the gene and protein expression of the pro-inflammatory cytokines IL-6 and TNF-α in tumor cells from the MelJuSo cell line in contact with the tested materials (Coll, Coll-C, Coll-Q, and Coll-CQ) and in untreated tumor cells grown on plastic (positive control).

**Figure 3:**
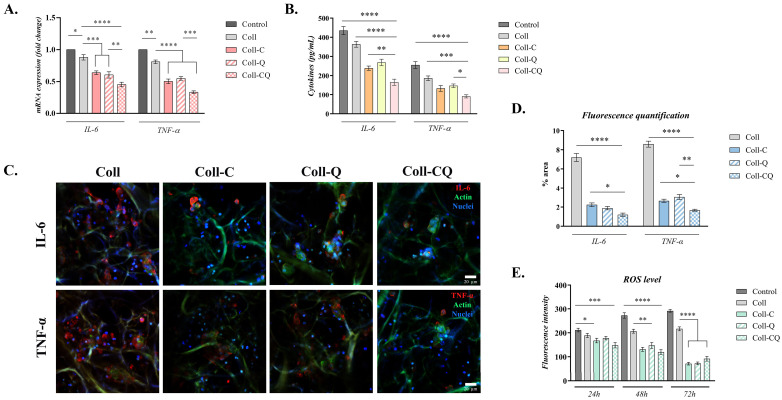
Evaluation of the anti-inflammatory and antioxidant potential of the delivery systems: (**A**) Anti-inflammatory effect of MDDS-C/Q revealed by evaluating the gene expression of pro-inflammatory markers in melanoma cells exposed to the treatment, 48 h post-seeding, compared to untreated tumor cells grown on plastic (control); (**B**) Protein expression level of pro-inflammatory molecules IL-6 and TNF-α in the culture medium of tumor cells found in contact with collagen-based sponges enriched with encapsulated flavonoids, compared to tumor cells grown on plastic (positive control), 48 h after seeding; (**C**) Fluorescence microscopy images showing protein expression of pro-inflammatory cytokines IL-6 and TNF-α labeled with Alexa Fluor 546-coupled antibodies (red), actin filaments labeled with phalloidin (green), and nuclei with Hoechst (blue) in melanoma cells found in contact with the materials supplemented or not with flavonoids. Scale bar 20 μm; (**D**) Fluorescence quantification of IL-6 and TNF-α protein expression (red fluorescence, labeled AF546) levels on all composites; (**E**) Fluorimetric analysis of ROS levels in the culture medium of melanoma cells seeded on the tested materials (Coll, Coll-C, Coll-Q and Coll-CQ), compared to cells grown on plastic (control). Statistical significance: * *p* < 0.05, ** *p* < 0.01, *** *p* < 0.001, **** *p* < 0.0001. Specific dosage of natural agents released from MDDS: 50 µM curcumin and 30 µM quercetin.

Thus, in Figure 3A, a statistically significant decrease in IL-6 and TNF-α expression can be observed in tumor cells under C/Q treatment, compared to the simple material (IL-6: *p* = 0.0002 for Coll-C; *p* < 0.0001 for Coll-Q and Coll-CQ, and in the case of TNF-α: *p* < 0.0001 for all flavonoid composites) and the positive control (*p* < 0.0001 for all materials with C and/or Q). A statistically significant difference was also observed between treatment with a single encapsulated nutraceutical agent and their simultaneous action. The most pronounced decrease in the expression of the pro-inflammatory markers was obtained in the case of Coll-CQ compared to Coll-C (*p* = 0.0014 for IL-6 and *p* = 0.0027 for TNF-α), and Coll-Q (*p* = 0.0068 for IL-6 and *p* = 0.0004 for TNF-α), suggesting an enhanced anti-inflammatory effect of the combined curcumin–quercetin formulation (CQ-enriched material).

Furthermore, protein expression of the two pro-inflammatory cytokines was evaluated by measuring their levels in cell culture media from melanoma cells found in contact with the tested materials (Figure 3B), as well as by immunolabeling coupled with fluorescence microscopy (Figure 3C), followed by fluorescence quantification (Figure 3D). In both cases, a significant decrease in IL-6 and TNF-α expression was recorded in melanoma cells treated with C/Q, either encapsulated alone or in combination.

Thus, as can be seen in Figure 3B, the protein expression levels of pro-inflammatory cytokines was significantly reduced in the presence of C/Q flavonoids encapsulated in MDDS, compared to the positive control (*p* < 0.0001 for IL-6 and *p* = 0.0003 for TNF-α), as well as compared to the collagen-based material without microcapsules (*p* < 0.0001 for IL-6 and *p* = 0.0005 for TNF-α). In general, the best result was obtained at the Coll-CQ level (*p* < 0.0001 for both cytokines), with significant differences also detected between the material with both nutraceutical compounds and the composites with a single encapsulated flavonoid (statistically: *p* = 0.0038 for IL-6 and *p* = 0.0251 for TNF-α in the case of Coll-CQ versus Coll-C; *p* = 0.0006 for IL-6 and *p* = 0.0189 for TNF-α in the case of Coll-CQ versus Coll-Q). Microscopy images also revealed a decrease in IL-6 and TNF-α expression in melanoma cells exposed to the C/Q composites, compared to the control material (almost 4–5× lower), suggesting that the encapsulated phytocompounds have an anti-inflammatory effect, more pronounced in the presence of both flavonoids (*p* = 0.0268 for Coll-CQ versus Coll-C for both markers and *p* = 0.0042 for Coll-CQ versus Coll-Q in the case of TNF-α) (Figure 3C,D). These results are in accordance with the aforementioned data and suggest a synergistic effect of C/Q and the enhanced anti-inflammatory potential of Coll-CQ.

### 3.4. Evaluation of Antioxidant Potential of MDDS-C/Q

The antioxidant potential of MDDS-C/Q complexes embedded in collagen-based materials was evaluated by performing a fluorimetric analysis to measure the levels of ROS secretion after 24, 48 and 72 h of contact between melanoma cells and the tested materials, compared to a positive control (tumor cells grown on plastic) (Figure 3E).

After 24 h, the level of ROS secretion remained approximately constant, with no significant differences between the composites with C/Q and simple Coll, but a statistically significant decrease was observed compared to the plastic control in the case of Coll-C (*p* = 0.026) and Coll-CQ (*p* = 0.0005) materials. After 48 h, the differences became more pronounced, and a statistically significant decrease in ROS release was detected in the case of composites with MDDS-C/Q compared to the control (*p* < 0.0001), but also compared to Coll (*p* = 0.0064 for Coll-Q, *p* = 0.0007 for Coll-C and Coll-CQ). Finally, after 72 h, the amount of ROS secreted was significantly reduced compared to both the control and the material without flavonoids (*p* < 0.0001), indicating the antioxidant potential of C/Q, but also the fact that it is maintained over time.

Therefore, reduced intensity of ROS release observed in the collagen-based composites, but also the relative increase over time of ROS secretion in the case of the positive control, indicates that MDDS-C/Q embedded in biomaterials have antioxidant potential, with a more pronounced effect in the presence of both flavonoids (Coll-CQ).

## 4. Discussion

Research regarding nutraceuticals has advanced in recent decades, allowing the identification and isolation of biologically active compounds from foods that can be used for therapeutic or preventive purposes to improve overall health and support the treatment of existing conditions. Flavonoids have become popular nutraceuticals, and in the case of melanoma, anti-tumor effects have been observed, but the identification of molecular targets has been difficult, with most studies unable to determine whether the effects of flavonoids stem from their direct action or represent indirect effects. Although flavonoids are extremely promising nutraceutical compounds, their molecular mechanism and multi-lateral nature (targeting multiple molecules simultaneously) require further investigation before advancing to translational or clinical studies [27].

A key aspect of this study is the evaluation of curcumin (C) and quercetin (Q) embedded in a collagen-based microcapsule system (Coll-MDDS), representing an innovative strategy for the sustained delivery of bioactive compounds in a physiologically relevant 3D environment. In this context, we assessed the effectiveness of the composites on melanoma cell viability and proliferation, as well as anti-inflammatory and antioxidant potential, for prospective applications in cancer therapy.

Considering that cell death is an important mechanism in anti-neoplastic therapy, the evaluation of the anti-tumor effect of MDDS-C/Q on malignant melanocytes (MelJuSo, a primary melanoma cell line in the vertical growth phase, therefore with invasive tendencies) was carried out through various viability and proliferation assays (MTT, LDH and LiveDead), as well as through the analysis at the gene and protein level of markers involved in apoptosis (Bcl-2, Bax, Caspase-3). It was observed that cell viability and proliferation were significantly reduced in the case of Coll materials supplemented with C/Q, and the best results were obtained for the composite with both bioactive compounds (Coll-CQ). Interestingly, the treated tumor cells had red nuclei while retaining green cytoplasmic staining, indicating that they were in a transient state of apoptosis, still metabolically active, but with compromised membrane integrity [28]. At the same time, a statistically significant increase in the pro-apoptotic markers Bax and Caspase-3 (cleaved) was recorded under the action of flavonoids, while the expression of the anti-apoptotic marker Bcl-2 was significantly reduced, suggesting that the data support activation of apoptotic pathways (a fact also highlighted by the formation of apoptotic bodies). The results obtained are consistent with studies found in the literature, with curcumin and quercetin affecting the viability and proliferation of melanoma cells mainly by arresting the cell cycle and inducing apoptosis, but also by inhibiting signaling cascades involved in tumorigenesis (e.g., PI3K/AKT and MAPK). The synergistic effect of the two flavonoids has also been reported, with the combined treatment being a more potent inhibitor of cell viability in melanoma [29]. For example, quercetin has been shown to exert anti-tumor activity in vitro in melanoma cell lines SK-MEL-28 and G-361 by reducing viability (IC_50_ ≈ 269–272 µM), inducing cell cycle arrest in the G0/G1 phase, mitochondrial depolarization, cytochrome-c release and apoptosis. It also negatively regulates FAK/paxillin/Akt signaling, which is involved in tumor progression [30]. Regarding the action of curcumin in melanoma, it affects viability also by arresting the cell cycle (in the G2 phase) and inducing apoptosis; it has been studied including in animal models, where it was found to significantly reduce tumor volume and weight compared to the control group (it also reduced lung nodules), the dose being between 25 and 50 mg/kg over a period of 10–20 days for murine models. Apoptosis rates varied, highlighting heterogeneity and possible non-apoptotic effects [31,32]. Interestingly, these two flavonoids show mechanistic synergy, for example in A375 melanoma cells suppressing the Wnt/β-catenin signaling pathway and targets of the apoptotic cascade at a dose of ~1.5–50 µM [29]. Although our current study does not include normal melanocytes as controls, previous work from our group demonstrated that curcumin and quercetin exert minimal cytotoxicity on human keratinocytes at concentrations effective against melanoma cells [25], suggesting some degree of tumor selectivity. The results are promising, but require further investigation, such as the inclusion of primary melanocytes or immortalized non-tumorigenic melanocyte lines to fully evaluate potential toxicity, therapeutic efficacy, and any melanin-related interference with assay readouts.

In addition, a decrease in the expression of pro-inflammatory markers IL-6 and TNF-α was observed at both the gene and protein levels, after the exposure of melanoma cells to the flavonoid-enriched microcapsule systems, as well as a decrease in ROS secretion over time, which indicated the anti-oxidant potential of the delivery systems supplemented with the tested flavonoids, these properties being important for tumor microenvironment (TME) modulation [33]. The results suggest that embedding flavonoids in the collagen-based system may allow for sustained exposure, potentially enhancing their bioactivity compared with free compounds. Although our study focused on downstream biomarkers (IL-6, TNF-α, and ROS), curcumin and quercetin are known to modulate key upstream signaling pathways. Curcumin inhibits NF-κB activation by blocking IκB kinase, thereby reducing the nuclear translocation of p65 and subsequent transcription of pro-inflammatory cytokines. Quercetin similarly suppresses NF-κB activation. Both compounds can also activate the Nrf2 pathway, promoting the expression of antioxidant enzymes such as HO-1 and SOD, which can explain the observed reduction in ROS levels. These mechanisms suggest that curcumin and quercetin may exert anti-inflammatory and antioxidant effects that contribute to a tumor-suppressive microenvironment, supporting their potential role as adjuvant anti-tumor agents [34,35].

Both curcumin and quercetin have been reported to act as anti-inflammatory agents, potentially suppressing viability and modulating the inflammatory TME that supports melanoma progression, mainly by inhibiting the NF-κB signaling pathway and decreasing the secretion of pro-inflammatory cytokines (e.g., IL-1β, IL-6, IL-8, TNF-α, COX-2). Singularly, curcumin affects the STAT3 signaling pathway, involved in inflammation and survival and is often overactive in melanoma, and inhibits invasion by modulating inflammation-induced MMP expression. It has also been found to sensitize tumor cells to immunotherapy and chemotherapy by reducing inflammatory resistance mechanisms. Quercetin, in turn, modulates the MAPK (ERK/JNK/p38) and PI3K/Akt signaling pathways, which are upstream of inflammatory cytokine expression, negatively regulates angiogenesis (targeting VEGF), and inhibits metastatic progression. The concomitant use of curcumin and quercetin shows synergy, by inhibiting the NF-κB and Wnt/β-catenin pathways, as well as potently suppressing pro-inflammatory mediators. In addition, the association with quercetin increases the bioavailability of curcumin, which enhances anti-tumor efficacy [33,34,35,36,37,38]. In short, previous studies have reported that curcumin and quercetin can modulate NF-κB- and Nrf2-related signaling pathways in various cancer and inflammatory models. Therefore, the reductions in IL-6, TNF-α, and ROS observed in the present study may be consistent with mechanisms involving modulation of these pathways. However, NF-κB, Nrf2, and their downstream effectors were not directly assessed in the current work; thus, these mechanisms should be considered literature-supported hypotheses rather than experimentally demonstrated findings.

Concerning the antioxidant effects observed in the present study, they may be related to mechanisms previously reported for curcumin and quercetin, including the modulation of Nrf2-associated antioxidant responses. According to studies found in the literature, melanoma is a redox-adaptive cancer, therefore tumor cells upregulate enzymes involved in the antioxidant processes (e.g., NRF2, HO-1, SOD, CAT, and GPX) to survive oxidative stress, with a cytoprotective effect. Sustained NRF2 activity may also promote invasion and resistance to treatment. In this context, although curcumin and quercetin may have an antioxidant effect, this is generally dose- and time-dependent, more precisely lower doses exert antioxidant effects (often through the activation of NRF2/HO-1 and related enzymes), while high doses or the accumulation over time of these flavonoids increase ROS secretion, cause GSH depletion and ROS-dependent cell death (apoptosis/necroptosis), thus having a biphasic effect. In the case of melanoma, pro-oxidant, cytotoxic effects have been more often reported, but the results obtained in this study revealed the antioxidant effect of flavonoids, possibly being dose-dependent [4,39,40,41,42]. Nevertheless, because Nrf2 signaling and antioxidant enzyme expression were not directly measured, the present data do not allow confirmation of these mechanisms.

In conclusion, both curcumin and quercetin affect the viability and proliferation of tumor cells by arresting the cell cycle (curcumin in the G2/M phase, and quercetin in the G1 phase), inducing apoptosis (by activating caspases), as well as by inhibiting several signaling cascades, the common one being the MAPK pathway. A synergistic effect of the two flavonoids has also been reported, with the combined treatment being a more potent inhibitor of melanoma cell viability. Regarding the anti-inflammatory and antioxidant effects, these represent important advantages for modulating the TME in order to reduce inflammation and oxidative stress that may influence melanoma tumor progression. However, the present study has several limitations that should be considered when interpreting the findings. One of them is that the molecular mechanisms underlying the observed anti-inflammatory and antioxidant effects were not directly investigated. While previous studies have implicated signaling pathways such as NF-κB, Nrf2, PI3K/Akt, MAPK, and Wnt/β-catenin in the biological activity of curcumin and quercetin, the current work assessed only downstream functional and molecular endpoints (IL-6, TNF-α, ROS, Bax, Bcl-2, and Cleaved Caspase-3). Therefore, any involvement of these signaling pathways should be regarded as hypothetical and requires confirmation through dedicated mechanistic studies. Another limitation of the present study is the use of a single melanoma cell line. Although MelJuSo cells represent a clinically relevant model of primary melanoma with invasive characteristics, melanoma is a highly heterogeneous disease comprising multiple molecular and phenotypic subtypes. Consequently, the biological responses observed in the present study may not fully reflect those of other melanoma cell lines or patient-derived tumors. Future studies should therefore validate these findings in additional melanoma models with diverse genetic backgrounds and metastatic potential to determine the broader applicability of the proposed delivery system. Additionally, no in vivo validation was conducted. Consequently, the current data do not provide information regarding pharmacokinetics, biodistribution, tissue penetration, local retention, systemic toxicity, or therapeutic efficacy in living organisms. Therefore, the observed biological effects should be considered preliminary and indicative of the potential of the delivery platform rather than evidence of clinical applicability. Future studies should incorporate relevant animal models and comprehensive safety evaluations to establish the translational potential of the proposed system.

Finally, while the current study focuses on the delivery system, without detailed mechanistic characterization, prior work from our group demonstrated that encapsulation enhances the stability, solubility, and controlled release of therapeutic agents compared with free compounds [26], indicating the potential of this microcapsule system to provide sustained exposure and enhance bioactivity relative to free compounds. This represents an innovative approach with translational relevance. These properties support a potential therapeutic advantage of the MDDS delivery system. Future work should include direct side-by-side comparisons of free and encapsulated compounds to further quantify their relative efficacy, as well as additional melanoma cell lines and pigmented and non-pigmented controls, to determine whether the observed antiproliferative and pro-apoptotic effects are selective for melanoma cells and to evaluate the safety profile of the encapsulated flavonoids. Furthermore, in vivo evaluations to validate the observed effects of curcumin and quercetin and to allow for more detailed mechanistic studies are necessary, in order to further assess the therapeutic potential of this delivery system. Curcumin and quercetin therefore represent extremely promising compounds, but further investigations are required to determine optimal dose and increase their bioavailability before their potential can be explored in translational and clinical studies [27].

## 5. Conclusions

The study highlights the enhanced anti-tumor effects of two nutraceutical compounds (curcumin and quercetin) on melanoma cell proliferation and survival, as well as their innovative encapsulation in microcapsules intended for gradual delivery and improved bioavailability, embedded in a collagen matrix, with a potential role in chemoprevention. The results demonstrate that sustained delivery of these flavonoids reduces melanoma cell viability, promotes apoptosis, and modulates inflammatory- and oxidative stress-related markers in vitro. While these findings support the potential of the proposed delivery platform for localized flavonoid administration, further studies in more complex experimental models, including in vivo investigations, are required to evaluate safety, efficacy, and translational relevance. Therefore, the current work should be considered a proof-of-concept study supporting the continued development of controlled release flavonoid delivery systems for melanoma-related applications.

## Figures and Tables

**Table 1:** Composition of the microcapsules with flavonoids.

Microcapsules	Sodium Alginate [g]	CMCNa [g]	Gelatin [g]	Curcumin [g]	Quercetin [g]
MDDS-C	0.2	0.05	0.5	0.008	0
MDDS-Q	0.2	0.05	0.5	0	0.016
MDDS-CQ	0.2	0.05	0.5	0.008	0.016

Reported per 20 mL solution.

**Table 2:** Collagen-based scaffolds with embedded microcapsules.

Biomaterials	Description
Coll	Material based on type II collagen, extracted from bovine skin
Coll-C	Collagen-based material enriched with curcumin microcapsules
Coll-Q	Collagen-based material supplemented with quercetin microcapsules
Coll-CQ	Collagen-based material enriched with curcumin and quercetin microcapsules

## Data Availability

The original contributions presented in this study are included in the article. Further inquiries can be directed to the corresponding author.
